# Development of Gluten‐Free Crispy Chicken With Vegetable Powder Coating

**DOI:** 10.1155/ijfo/4928193

**Published:** 2025-09-08

**Authors:** Kivilcim Ates Cakiroglu, Elif Savas, Zeynep Kilci, Aslan Deniz Karaoglan, Ramazan Ulku Cetin

**Affiliations:** ^1^ Department of Research & Development, HasTavuk Company, Balikesir, Turkey; ^2^ Department of Food Engineering, Balikesir University, Balikesir, Turkey, balikesir.edu.tr; ^3^ Department of Food Processing, Bandirma Onyedi Eylul University, Balikesir, Turkey, bandirma.edu.tr; ^4^ Department of Industrial Engineering, Balikesir University, Balikesir, Turkey, balikesir.edu.tr

**Keywords:** coating adhesion ratio, dietary fiber, DSC, gluten-free crispy chicken, RSM

## Abstract

There is a growing interest in newly formulated meat products, such as crispy chicken, that are both delicious and quick to prepare, especially when coated with high‐fiber and/or gluten‐free ingredients. In this study, the potential for producing gluten‐free crispy chicken was investigated by using corn fiber for predusting, corn flour in liquid coating formulations, and a mixture of dried vegetable powder and buckwheat flour in the outer coating. The effects of these formulations on the textural and physicochemical properties of the chicken were analyzed. The liquid coating components were optimized using response surface methodology (RSM) to achieve ideal viscosity, maximize surface coverage, and minimize deformation during cooking. For this formulation and the control, key parameters were analyzed, including coating adhesion, cooking yield, coating thickness, fat absorption, dietary fiber content, energy, crude protein, carbohydrate, ash, moisture, gluten content, texture, thermal behavior (via differential scanning calorimetry), hydroxymethylfurfural (HMF), thiobarbituric acid reactive substances (TBARS), and microbial quality (*Salmonella* spp., coliforms, *Pseudomonas* spp., yeast and mold, and total mesophilic aerobic bacteria) over 11 days of storage, as well as sensory attributes such as color.

## 1. Introduction

Poultry products are among the most popular and widely consumed foods worldwide. These products are generally perceived by consumers as healthy and nutritious, providing high‐quality protein with relatively low‐fat content, while also being rich in unsaturated fatty acids [1, 2]. Factors such as population growth, the absence of cultural and religious restrictions, low production costs, and greater affordability compared to red meat have contributed to the increasing demand for poultry meat, which is expected to continue as a growing trend. Furthermore, various processing methods and the development of new products have contributed to the rising consumption of poultry. In particular, coated chicken products, known for their golden color, crispiness, and taste, are popular across all age groups due to their quick preparation and convenience for hot serving [3]. However, despite the growing demand for coated meat products, there are concerns regarding their health implications. Food safety risks associated with processed meat products and the high fat absorption during frying are key factors that contribute to these concerns [4]. High fat consumption can lead to sudden increases in blood pressure and cholesterol levels. Therefore, developing food products with low‐fat content by reducing fat absorption is critical in mitigating the incidence of health issues such as obesity and high cholesterol levels. Consequently, the increasing demand for healthier and safer foods has driven the development of formulations with natural and functional ingredients for producing coated meat products [5, 6].

In the industrial production of coated meat products, wheat flour and starch are commonly used. This is primarily due to the presence of gluten, the wheat protein responsible for creating an elastic structure that ensures the coating adheres to meat products. However, there are some negative consequences associated with this usage. Although wheat flour and starch offer advantages such as binding fat and water and increasing protein content, they can also lead to various health issues. Celiac disease is the most well‐known gluten‐related disorder, but nonceliac gluten sensitivity, dermatitis herpetiformis (Duhring’s disease), wheat allergy, gluten ataxia, and irritable bowel syndrome are also associated with gluten consumption [7–9]. Celiac disease is a condition affecting the human small intestine due to impaired protein digestion and is estimated to affect 1 in 100 people worldwide. It is an autoimmune disorder triggered by the consumption of gluten‐containing foods, leading to intestinal damage and symptoms such as abdominal pain, diarrhea, weight loss, fatigue, and nutrient malabsorption. The only effective treatment for this disease is a strict lifelong gluten‐free diet, which must be rigorously followed by patients [10]. The dietary needs of individuals with celiac disease have increased the demand for gluten‐free products. However, celiac patients often face difficulties accessing gluten‐free products due to gluten contamination in many processed foods, the limited availability of basic gluten‐free products, and the high cost of gluten‐free alternatives [11, 12].

Although meat products are rich in protein, they are deficient in dietary fiber [13]. Therefore, the use of dried vegetables, which are rich in dietary fiber, in meat processing has several advantages. These include improving the health profile and nutritional value of the products, reducing total fat content, enhancing functional parameters such as water holding capacity (WHC), increasing product yield by reducing cooking loss and cost, and improving textural properties and storage stability [14]. Additionally, the inclusion of alternative grains in meat product formulations can further enhance functionality, quality, and cost‐effectiveness. Corn flour is considered one of the most suitable flours for developing gluten‐free products due to its mild flavor, easily digestible carbohydrate content, low prolamin content, and hypoallergenic properties [15, 16]. Furthermore, the zein protein found in corn flour helps retain moisture in the product and prevents the absorption of frying oil [4]. Buckwheat, on the other hand, is a gluten‐free grain source rich in nutritional value, including protein, resistant starch, dietary fiber, D‐chiro‐inositol, vitamins, and minerals [17]. It is also known to have higher antioxidant activity and functional properties compared to wheat [18]. In meat product coatings, the response surface methodology (RSM), which is one of the most commonly used experimental design techniques for mathematical modeling, is frequently employed [19–21].

Crispy chicken is a battered, coated, and fried meat product characterized by a dry, crunchy outer crust and a soft, tender inner portion. The final texture of crispy chicken largely depends on the liquid and dry coating applied after processing, as well as the moisture content and the composition of the coating materials. The outer layer of crispy chicken is exposed to high temperatures during frying, which dehydrates the coating and creates a crispy crust, an important sensory quality attribute. A review of the literature reveals several studies on gluten‐free chicken products prepared with wheat, corn, soybean and rye flours [3], chia and quinoa flours [6] amaranth, buckwheat and einkorn [22] cellulose, egg powder, whey powder, pectin, and different combinations, all of them with gluten‐free wheat flour [23]. However, no studies have been found that utilize corn fiber in the predusting process, corn flour in the liquid coating, and a mixture of high‐fiber vegetable powders, corn fiber, and buckwheat flour in the dry coating for the production of crispy chicken. Preparing gluten‐free meat products using cereal sources and dried vegetables is an emerging area of interest for researchers and the industry. In general, the adhesion of coatings in these product groups is achieved using eggs. Since eggs can cause allergic reactions, particularly among children, this study replaces eggs and gluten‐containing starch‐based liquid coatings with corn flour. The aim is to develop a gluten‐free nugget containing dietary fiber as an alternative to wheat flour.

## 2. Materials and Methods

### 2.1. Material

The chicken breasts used in the production of gluten‐free crispy chicken were sourced from the slaughterhouse of Hastavuk Gıda Tarım Hayvancılık Sanayi ve Ticaret A.Ş. (Balikesir, Türkiye). The corn fiber (Vanikoy Fibrigem FBR B + PWDR) used for predusting was obtained from Cargill (Minnesota, United States), while the corn starch used in the liquid coating was supplied by Dr. Oetker (İstanbul, Türkiye). The buckwheat flour and corn semolina used in the dry coating were procured from İpek Değirmen (Aksaray, Türkiye), and the salt was provided by Billur Tuz (İzmir, Türkiye). The dried and ground vegetable powders (oyster mushroom, spinach, carrot, onion) were obtained from Kurucum (Isparta, Türkiye). In determining the mixture ratios of the dried vegetable powders, the results of the patent application filed by HasTavuk R&D Center on 12.06.2023 under patent number 2023/006823 were utilized. The ingredients and their ratios used in the mixture are presented in Table 1. All analytical reagents used in this study were purchased from Merck (Darmstadt, Germany).

**Table 1:** Experimental design.

**Experiment no.**	**Corn starch (%)**	**Salt (%)**
	**X**_1_	**X**_2_
1	25	1
2	50	1
3	25	3
4	50	3
5	25	2
6	50	2
7	37.5	1
8	37.5	3
9	37.5	2

### 2.2. Methods

#### 2.2.1. Experimental Design

**Figure 1:**
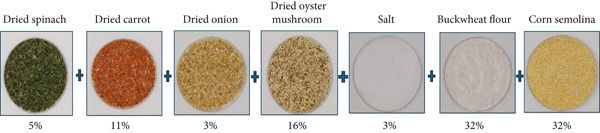
Coating/crumbs’ dry vegetable mix contents.

**Figure 2:**
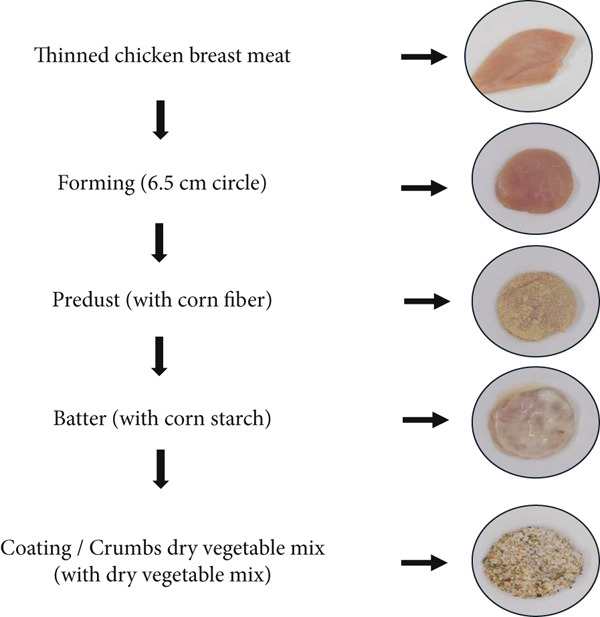
Production method flowchart.

In the production of gluten‐free crispy chicken, chicken breast meat that had completed the rigor mortis process and had been rested for 24 h at 1°C–4°C was used. The chicken breasts were manually cut into circular shapes with a diameter of 6.5 cm, and each portion was adjusted to weigh 33 g (Figure 1). These shaped chicken breasts were predusted with insoluble corn fiber, ensuring that the entire surface was coated (Figure 2). Since the liquid coating is the most important parameter affecting the adhesion efficiency of the dry coating in crispy chicken production, a model consisting of nine experimental trials was developed using Minitab 16.0 software (2010 Minitab Inc.) and RSM to determine the appropriate corn starch (*X*_1_) (25, 37.5, 50), salt (*X*_2_) (1, 2, 3), and water ratios and to optimize the viscosity of the mixtures (Table 1). Factor levels in percentage are selected as 25, 37.5, and 50 for *X*_1_ and 1, 2, and 3 for *X*_2_, respectively. Water was not selected as a separate factor because the total mixing ratio should be 100%. It is the water ratio that will complete the sum of *X*_1_ and *X*_2_ in each experiment to 100%, and it is the dependent variable calculated based on the values of *X*_1_ and *X*_2_. The significance level is used as 0.05 in the statistical analysis. Trials were performed in three replicates, and the results are given as mean ± SD.

Liquid coatings containing wheat starch available in the market were selected as the control sample, and the viscosity value of approximately 15 mPa·s was considered as the control group for the experimental design in this study. In preliminary tests, results significantly different from an average of 15 mPa were obtained when using less than 25% or more than 50% corn starch, so the range was selected as 25%–50%. It is known that salt increases viscosity when it interacts with water. Values close to those specified in the “Turkish Food Codex Notice On Meat, Prepared Meat Mixtures, and Meat Products” were selected to investigate the effect on viscosity. The required explanations are added into the text. The viscosity values of the mixtures, prepared according to the formulations in the experimental design to ensure coating adhesion, were adjusted to be close to those of the control sample. In the final stage, after applying the dry coating according to the formulation of the final design, the analyses were conducted. In order to determine their storage stability, the samples were stored in polyethylene locked refrigerator packages at +4°C for 11 days.

#### 2.2.2. Chemical Composition and Energy

##### 2.2.2.1. Determination of Protein Content

Homogenized and weighed crispy chicken samples were placed into Kjeldahl distillation tubes, and H_2_SO_4_ (*d* = 1.84 g/mL) was added to facilitate the conversion of organic nitrogen to ammonium sulfate by heating the mixture in the system. After the distillation was completed, the tube was allowed to cool, and following the cooling process, distillation commenced. Distillation continued until ammonia gas was collected in an Erlenmeyer flask containing a 4% boric acid solution. The collected ammonia was titrated with a 0.1 N H_2_SO_4_ solution, and the total nitrogen content was first determined using Equation (1). Subsequently, the total protein content was calculated as a percentage using the correction factor described in Equation (2) (method 968.06) [24]:

(1) Total nitrogen%=Vs−Vb×N×1.4007/W

(2) Protein %=total nitrogen %×6.25

where *V*_b_ is the blank titration volume (milliliter), *V*_s_ is the sample titration volume (milliliter), *N* is the normality of H_2_SO_4_, and *W* is the sample weight (gram).

##### 2.2.2.2. Determination of Fat Content

Three grams of homogenized and preweighed crispy chicken samples was dried in an oven (Geotechnical Testing Equipment, United Kingdom) at 105^°^C ± 2^°^C until a constant weight was achieved in approximately 4 h. After cooling in a desiccator, the samples were weighed. The Soxhlet apparatus was then prepared for extraction, and the samples were placed into extraction tubes. Hexane was used as the solvent, and the extraction process was carried out for 4–6 h. After the solvent was removed using a rotary evaporator, the drying process was repeated to calculate the residue remaining in the extraction tube. The fat content was then calculated as a percentage using Equation (3), as presented below [25]:

(3) Fat %=weight of extracted fat/weight of sample×100

##### 2.2.2.3. Determination of Ash Content

Homogenized and preweighed crispy chicken samples were dried in an oven (Geotechnical Testing Equipment, United Kingdom) at 105^°^C ± 2^°^C until a constant weight was achieved, followed by cooling in a desiccator and subsequent weighing. A constant weight is considered to be achieved when the difference between two consecutive weighings is less than 0.001 g. The organic residues in the ash crucible were incinerated in a muffle furnace (Sante Furnace Technical Co. Ltd., China) at 550^°^C ± 2^°^C, and after cooling in a desiccator, the samples were weighed again. The prepared sample was placed in an ash crucible and incinerated for 4–6 h until complete ashing, during which all organic matter was eliminated. After cooling, the final weight was recorded, and the ash content was calculated as a percentage using Equation (4), as shown below [26]:

(4) Total ash %=weight of crucible with ash−weight of empty crucible/weight of sample×100

##### 2.2.2.4. Determination of Moisture Content

A 5‐g sample of crispy chicken was weighed and dried in an oven (Geotechnical Testing Equipment, United Kingdom), at 105^°^C ± 2^°^C until a constant weight was achieved. The sample was then cooled in a desiccator, and the final weight was determined. The percentage of moisture content was calculated using Equation (5), as presented below [27]:

(5) Moisture content %=initial weight−final weight/sample weight×100

##### 2.2.2.5. Determination of Total Dietary Fiber

The dietary fiber content of the samples was analyzed using the Megazyme total dietary fiber kit (Megazyme International Ireland Ltd., Wicklow, Ireland), following the standard methods [28, 29], which involve the use of *α*‐amylase, protease, and amyloglucosidase enzymes. The weighed sample was gelatinized with heat‐resistant *α*‐amylase at 95°C–100°C to hydrolyze digestible starch. Then, enzymatic digestion with protease and amyloglucosidase at 60°C was performed to remove digestible proteins, resulting in the collection of the insoluble dietary fiber fraction, which included insoluble salts and undigested proteins. To precipitate the soluble fraction of the dietary fiber, 95% ethanol was added to the collected filtrate and left at room temperature for 1 h. This allowed for the collection of the soluble dietary fiber fraction along with minerals and undigested proteins. The total dietary fiber content was calculated by summing the amounts of soluble and insoluble dietary fiber. The equation used for the calculation is presented in Equation (6):

(6) TDF %=dried residue weight−ash weight/sample weight×100

##### 2.2.2.6. Determination of Carbohydrate Content

After completing the analyses for moisture, ash, protein, fat, and fiber content, the carbohydrate content was calculated using Equation (7), as presented below [30]:

(7) Carbohydrate=100−moisture+ash+protein+fat+fiber

##### 2.2.2.7. Determination of Energy Content

The total energy value (kcal/100 g) was calculated by multiplying the amounts of protein, fat, and carbohydrates by their respective energy equivalents. The calculation was performed using Equation (8), as presented below [27]:

(8) Energy=protein g×4+fat g×9+carbohydrate g×4

##### 2.2.2.8. Gluten Content

The gluten content was analyzed using the AgraQuant Gluten G12 kit, which is the currently accepted method for detecting gluten in foodstuffs. A 0.25‐g specimen was placed in a tube, and 2.5 mL of extraction solution was added. The mixture was incubated (Nuve, Turkey) at 50°C for 40 min, followed by the addition of 80% ethanol, and then rotated for 60 min for extraction. The extracts were subsequently centrifuged (MKE, China) for 10 min at 2000 × *g*, at 20^°^C ± 2^°^C. The wells were washed five times with the wash buffer. Afterward, 100 *μ*L of the conjugate was added to each well, incubated for 20 min at 20^°^C ± 2^°^C, and then removed. The washing step was repeated five times. Next, 100 *μ*L of substrate was added to the well and incubated in the dark for 20 min. Finally, 100 *μ*L of stop solution was added to each well, and absorbance (Shimadzu, Japan) was measured at 450 nm. A standard solution of 4–200 ppm gluten was used [31].

##### 2.2.2.9. Determination of Mineral Components

The mineral components in the products were determined using inductively coupled plasma mass spectrometry (ICP‐MS) (Thermo iCAP RQ ICP‐MS). The homogenized samples were treated with 65% HNO_3_ and 50% H_2_O_2_, followed by incineration, and then injected at room temperature to determine the amounts of iron, calcium, magnesium, and manganese [32]. Microwave settings are determined as 70°C/15 min, 85°C/10 min, 105°C/10 min, 70°C/15 min, 110°C/5 min, 120°C/5 min, and 130°C/5 min.

#### 2.2.3. Determination of Quality Characteristics

##### 2.2.3.1. Determination of Coating Adhesion Ratio (CAR)

Chicken breast samples were weighed prior to the liquid coating process and then dipped into the liquid coating sauce and held for 10 s to drain before being weighed again. The CAR was calculated using Equation (9), as presented below [33]:

(9) CAR %=weight of coated sample−weight of sample before coating/weight of coated sample×100

##### 2.2.3.2. Determination of Cooking Yield (CY)

The CY of the chicken breast samples was calculated by recording the weights before and after cooking, following the method described by [34], and using Equation (10), as presented below:

(10) CY %=cooked sample weight/raw sample weight×100

##### 2.2.3.3. Determination of WHC

A 1‐g sample was placed in filter paper and centrifuged (Nuve Nf 1200R) at 1500 × *g* for 4 min, at 25^°^C ± 2^°^C. The sample inside the filter paper was then dried overnight at 70°C [35]. The WHC was calculated using Equation (11) as presented below:

(11) WHC %=weight of filter paper+sample−weight of filter paper+postdrying weight/initial sample weight×100

##### 2.2.3.4. Determination of Absorbed Fat Content (AFC)

The amount of fat absorbed during deep frying of crispy chicken samples will be determined as a percentage based on the difference in fat content between the raw sample, coated before frying, and the final product after frying [36]. The AFC was calculated using Equation (12) as presented below:

(12) AFC %=fat content determined in the final product after frying/fat content in the raw sample coated with outer layer before frying×100

#### 2.2.4. Color Analysis

The color analysis of the samples was conducted using a Hunter Lab colorimeter (MiniScan EZ 45/0 LAV), based on the *L*∗ (lightness–darkness), *a*∗ (redness–greenness), and *b*∗ (yellowness–blueness) color system. Hue is defined as how a color is perceived and named, using the colors of the rainbow (red, orange, green, blue, etc.). Chroma, also known as saturation, describes the vividness or dullness of the color [37].

#### 2.2.5. HMF (5‐Hydroxymethylfurfural) Analysis

The HMF content of the samples was determined according to Winkler’s shiny and angular metric method. The dried and milled samples (2.5 g) were weighed into a tube and homogenized in 10 mL of n‐hexane for 1 min at 1500 rpm. The samples were then incubated at 40°C for 30 min and centrifuged in a 3K30 centrifuge (Sigma 4‐16KS, Germany) at 4500 × *g* for 5 min, at 4°C. The supernatant was removed to separate the lipids. The defatted samples were air‐dried and homogenized using an Ultraturrax (IKA T25, Germany) in 10 mL of water for 1 min before being transferred to a 25‐mL volumetric flask. The contents of the flask were thoroughly mixed with 0.5 mL of Carrez I solution and allowed to rest for 2 min, and then, 0.5 mL of Carrez II solution was added and mixed in the same way to remove coextractives. The flask was filled to the final volume with distilled water, and the solution was filtered using Whatman 42 paper. Two test tubes were prepared: one containing 2 mL of the filtrate and 5 mL of 10% p‐toluidine and the other with 1 mL of distilled water as a reference solution, while 1 mL of 0.5% barbituric acid solution was added to the other test tube as a sample solution. The maximum absorbance of both tubes was measured at 550 nm using a spectrophotometer [38].

#### 2.2.6. Differential Scanning Calorimetry (DSC) Analysis

The thermal properties of the products, characterized by denaturation and glass transition points, were determined using DSC (DSC 702, HITACHI High‐Tech Corporation, Japan) following two methods [39]. For this purpose, a sample of 20 ± 1 mg was placed into aluminum pans, which were hermetically sealed and then inserted into the device. To determine the denaturation temperatures, heating parameters from +30°C to +100°C at a rate of 5°C per minute were applied.

The glass transition temperatures (Tg) were determined using the following process steps: cooling from +30°C to −80°C at 5°C per minute (hold: 15 min), heating from −80°C to +30°C at 5°C per minute (hold: 1 h), cooling from +30°C to −80°C at 5°C per minute (hold: 15 min), and heating from −80°C to +30°C at 5°C per minute (hold: 30 min). Nitrogen gas was used in the analyses to create an inert atmosphere.

#### 2.2.7. Texture Profile Analysis (TPA)

TPA was conducted using a TA‐XT Plus texture analyzer (Stable Micro System, United Kingdom) on each sample, with two analysis samples prepared from each and measurements taken at three different points for each sample. The results are presented as the average of these values. For the analysis, 10∗10 mm crispy chicken pieces were compressed using a 36‐mm diameter stainless steel cylindrical probe at a test speed of 3 mm/s. Hardness was expressed in Newtons as the maximum force required to deform the sample during the first compression. Brittleness was expressed in millimeters as the maximum force required to fracture the sample. The hardness value was obtained from the first compression curve. Elasticity was defined as the rate at which the food returned to its original shape after the first compression [40].

#### 2.2.8. Microbiological Analysis

Each crispy chicken sample was opened from its package inside a laminar flow hood next to a Bunsen burner. A total of 10 g was cut from three different areas of each sample and placed into sterile stomacher bags, followed by the addition of 90 mL of sterile peptone water. The mixture was homogenized for 60 s in a stomacher device and then serially diluted in 9‐mL peptone water solutions. Each dilution was mixed for 10 s using a vortex. After the preliminary preparation, 1 mL of the dilutions was plated onto the following media: plate count agar (PCA) for total mesophilic aerobic bacteria (TMAB) analysis [41], dichloran rose bengal chloramphenicol agar (DRBC) for yeast and mold analysis [42], and violet red bile agar (VRBA) for coliform bacteria analysis [43]. The samples were spread onto petri dishes using a Drigalski spatula. The inoculated petri dishes were incubated at 37°C for 48 h, 25°C for 120 h, 37°C for 48 h, and 25°C for 48 h, respectively. The results were expressed as colony‐forming units per gram of sample (cfu/g). For *Salmonella* spp. analysis, after the preliminary preparation, plating was performed using RAPID *Salmonella* agar for isolation. This medium is used for the detection of *Salmonella* spp. based on C8‐esterase activity (preidentification) and to distinguish *Salmonella* spp. colonies from other Enterobacteriaceae by *β*‐glucosidase activity. After incubation, approximately 10 *μ*L was taken from the pre‐enrichment medium using a sterile loop and plated onto RAPID *Salmonella* agar using the streak plate method. The petri dishes were incubated at 38°C for 24 h, and the results were expressed as detected/not detected [44].

#### 2.2.9. Sensory Evaluation

Sensory evaluations were conducted with a panel of 33 voluntary participants using a 10‐point scale. The samples were presented to the panelists in standard portions, deep‐fried at 180°C for 4 min on all sides, and labeled with random three‐digit codes. Panelists were asked to evaluate the crispy chicken samples based on external appearance, crispiness, oiliness, coating quality, overall flavor, and aroma characteristics using a 10‐point hedonic scale (10: like extremely, 1: dislike extremely) [45].

#### 2.2.10. Statistical Analysis

The experimental design was conducted by using Response Surface Face Centered Design by the aid of the Minitab statistical package. Selection of ingredient ranges used in the formulation (factor levels) was performed by the results of pre‐experiments. The statistical analysis of the data obtained from the physical, chemical, microbiological, and sensory analyses of the samples was performed using the Minitab statistical package. Analysis of variance (ANOVA) was conducted to determine whether there were significant differences between the samples in terms of physical, chemical, microbiological, and sensory properties. Comparisons of variation sources that showed significant differences at *p* < 0.05 or *p* < 0.01 were performed using the Tukey test.

## 3. Results and Discussion

### 3.1. Mathematical Modeling and Optimization

In this study, RSM was used for mathematical modeling. The experimental design and the measured responses are provided in Table 2.

**Table 2:** Measured viscosity values for the experimental design.

**Experiment no.**	**Corn starch (%)**	**Salt (%)**	**Viscosity value (mPa·s)**
	*X*_1_	*X*_2_	*Y*
1	25	1	4.6
2	50	1	27.1
3	25	3	5.9
4	50	3	31.1
5	25	2	5.4
6	50	2	30.8
7	37.5	1	8.2
8	37.5	3	9.5
9	37.5	2	8.9

When examining the correlation between the factors and responses presented in Table 2, it is evident that the apparent viscosity values of the batter solutions are significantly influenced by the corn starch concentration (*r* = 0.921) (*p* < 0.05). However, the same effect was not observed for the salt concentration (*r* = 0.083) (*p* > 0.05). Table 2 shows that the highest viscosity value was found in the model containing 50% corn starch and 3% salt. In nugget production, the viscosity of the liquid coating material is directly related to the coating composition. This phenomenon, known as the plastic effect, generally arises from the interaction of starch and protein molecules in the coating material. This effect is explained by the restricted mobility of polymer chains that absorb different amounts of water due to entanglements in the polymer chains [46].

Preliminary trials are conducted for mathematical modeling, and a quadratic regression model is derived for the response values of viscosity. The Minitab statistical package is used for fitting the regression models and performing the significance tests. Equation (13) shows the fitted regression model [21]:

(13) Y=47.66673.16131.10.0551−X1+X2+X12

**Figure 3:**
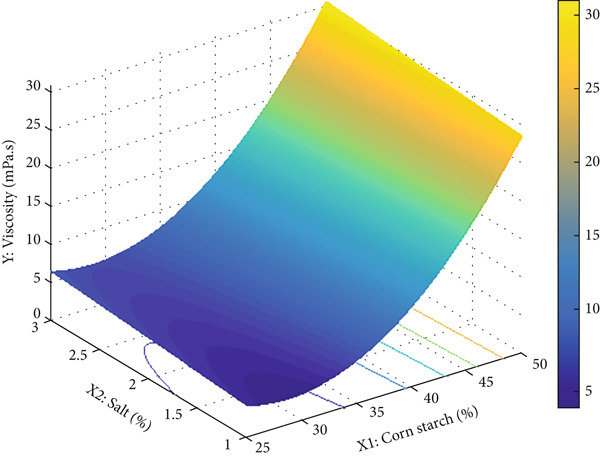
Response surface of viscosity.

**Figure 4:**
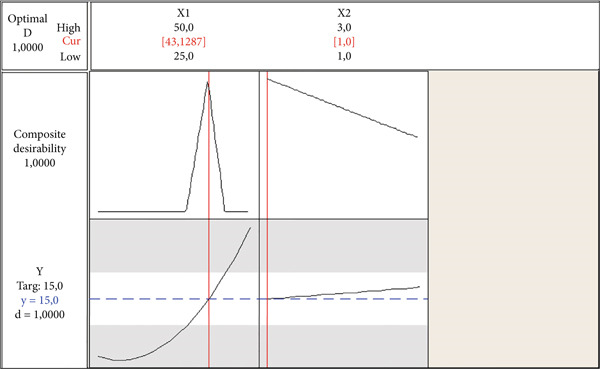
RSM optimization plot.

The *R* ^2^ for the regression model is calculated as 99.58%, which means that these factors are sufficient for representing the response. Also, the ANOVA is conducted, and the *p* value is calculated as 0.00 for the regression model, which means the model is significant and can be used for optimization. Additionally, the three‐dimensional graphical representation of the regression model given in Equation (13) is presented in Figure 3, while the RSM optimization plot obtained from the Minitab Response Optimizer Module is shown in Figure 4.

According to the results presented in Figure 4, the optimum factor levels for corn starch (percentage) and salt (percentage) are calculated as 43.1287% and 1%, respectively, and the response value for the viscosity is predicted as 15 mPa·s. The confirmation test for the optimum factor levels is also conducted, and the viscosity is measured as 15.4 mPa·s. This result indicates that the viscosity could be predicted with a very low error of 2.7%.

### 3.2. Nutritional Value and Energy

Chicken breast meat shaped into equal portions using molds was dipped into a liquid coating prepared with 43.12% corn starch, 3% salt, and water, which was determined to be the optimum point based on the viscosity values measured in the RSM mathematical model. The chemical composition and energy values of the raw and cooked samples, which were coated using the dry mixture provided in Figure 1, are presented in Table 3. Upon reviewing Table 3, it can be observed that the crude protein content ranged from 17.11% to 22.80%, crude fat from 0.58% to 4.76%, carbohydrate from 0.00% to 21.61%, ash from 1.04% to 1.82%, and moisture from 47.77% to 75.57%. The energy values ranged from 96.46 to 217.00 kcal/100 g, dietary fiber from 0.00% to 4.75%, calcium content from 60 to 207 mg/kg, magnesium from 230 to 342 mg/kg, and potassium from 2470 to 4022 mg/kg. No gluten was detected in any of the samples.

**Table 3:** Chemical composition and energy values of chicken breast (BM), coated raw chicken breast (UCBM), and coated cooked chicken breast (CCBM) samples.

**Parameter**	**BM**	**UCBM**	**CCBM**
Protein (%)	22.80 ± 0.56^a^	17.11 ± 0.43^c^	19.76 ± 0.49^b^
Fat (%)	0.58 ± 0.02^b^	4.59 ± 0.16^a^	4.76 ± 0.49^a^
Carbohydrate (%)	0.00 ± 0.01^c^	8.48 ± 0.16^a^	2.61 ± 0.17^b^
Ash (%)	0.04 ± 0.03^c^	0.33 ± 0.07^b^	0.82 ± 0.09^a^
Moisture (%)	75.57 ± 0.51^a^	63.68 ± 0.58^b^	47.77 ± 0.49^c^
Energy value (kcal/100 g)	96.46^c^	153.00^b^	217.00^a^
Dietary fiber (%)	0.00 ± 0.00^b^	4.75 ± 0.39^a^	4.32 ± 0.36^a^
Gluten content (g/100 g)	0.00 ± 0.00^a^	0.00 ± 0.00^a^	0.00 ± 0.00^a^
Calcium (mg/kg)	60 ± 0.98^b^	180 ± 22^a^	207 ± 26^a^
Magnesium (mg/kg)	230 ± 1.11^b^	341 ± 16^a^	342 ± 16^a^
Potassium (mg/kg)	2470 ± 187^b^	4022 ± 251^a^	3854 ± 240^a^

*Note:* Values are from average of three determinations. Means in rows followed by different letter(s) are significantly different (*p* < 0.05).

Abbreviations: BM, chicken breast meat; CCBM, cooked vegetable coated breast meat; UCBM, uncooked vegetable coated breast meat.

The higher protein value in the uncoated chicken breast compared to the coated samples is due to chicken meat being the primary source of protein. In the coated samples, the vegetables included in the calculations, and consequently their carbohydrate components, resulted in comparatively lower protein percentages. It is observed that the values of crude protein, fat, carbohydrates, ash, and energy are higher in the cooked samples compared to the raw ones. The water present in the raw samples is lost during the cooking process. The differences in moisture content between the samples are attributed to these relative changes. In fried coated products, the coating forms a layer on the product’s surface, preventing moisture loss due to evaporation and modifying the surface to prevent further oil penetration into the product. For this reason, moisture content is a significant quality criterion in fried products [47]. While no carbohydrates were detected in the uncoated chicken breast, their presence in the vegetable‐coated samples is attributed to the vegetables used in the coating, the precoating process, and the liquid batter. The vegetables used in the coating introduced dietary fiber, which is absent in chicken meat, to both raw and cooked samples. The slight decrease in dietary fiber content in the cooked samples is thought to be caused by the loss of coating material into the cooking medium during frying. In a study where amaranth, buckwheat, and einkorn flour and their mixtures were used as coating materials, the fat content of chicken nuggets was reported to range between 5.16% and 7.16%, protein content between 12.51% and 13.29%, and ash content between 1.62% and 1.80% [22]. Similar findings were also observed in a study by [48], which explored gluten‐free meatballs produced with flaxseed and coconut flour.

### 3.3. Physical Quality Characteristics

The results of some quality characteristics for the crispy chicken samples coated with the coating material containing the optimum component concentrations are presented in Table 4.

**Table 4:** Some quality characteristics of cooked chicken breast samples coated with vegetable powder.

**Quality characteristics**	**Value**
Coating adhesion ratio (%)	23.41 ± 0.050
Cooking yield (%)	81.63 ± 0.018
Cooking lost (%)	18.37 ± 0.018
Moisture content in cooked samples (%)	42.87 ± 1.80
Water holding capacity (%)	35.00 ± 0.017
Coating thickness (mm)	1.82 ± 0.235
Absorbed fat content (%)	14.73 ± 0.010

One of the critical factors influencing the final quality of coated products is the adhesion ratio of the coating material to the product being coated. It has been noted that flours with high protein content and hydrocolloids of different characteristics added to the coating formulations affect the CAR [49]; however, it is proposed that the primary structure providing adhesion originates from the flour added to the coating formulation [50]. Flour is a crucial coating material due to its plasticity, which results from its gluten and starch content. Studies have reported that the CAR of raw chicken nuggets dipped in coating formulations prepared with different flours, such as wheat, corn, soy, and rye, varies between 11.53% and 14.28% [3]. The high CAR determined in this study is thought to be due to the starch present in the wet coating material. In another study where wheat, soy, and rice flour were used as coating components in chicken nuggets, the highest CAR was detected with soy flour [51].

CY is an indicator of the adhesion ability of the coating to the surface during cooking, and as the yield increases, the economic value of the product also increases. Similarly, an increase in cooking loss negatively impacts the economic value of the product. In a study investigating the effects of different starch types on the CY of chicken nuggets, it was reported that tapioca starch provided better results compared to wheat and corn starch [52].

One of the sensory attributes sought in coated products such as chicken nuggets is juiciness. During cooking, coating components act as a natural barrier, reducing the loss of water from the meat and thus helping to maintain the juiciness of the product. A literature review on the moisture content of coated chicken nuggets reports values of 60.19% when coated with wheat, 59.50% with corn, 59.39% with soy, and 61.40% with rye [3]. It has been noted that the moisture content of chicken nuggets enriched with various proportions of pea flour ranged between 52.80% and 61.20% [53].

The moisture value determined for the crispy chicken sample in this study is comparatively lower than the values reported in the literature. Undoubtedly, the temperature increase during frying is a crucial factor that enhances moisture loss in coated products [54]. During the heating process, the gelatinization of starch‐containing gluten coatings due to moisture absorption helps retain moisture in coated products like crispy chicken. However, dietary fibers, which are the main components of vegetable mixtures, have significantly lower gelatinization potential compared to starch. This results in reduced moisture retention in the chicken during the frying process, leading to lower moisture values in the final product. A review of the literature on coating thickness after cooking shows values close to those measured in this study, with coatings made from soy flour at 1.28 mm and rye flour at 1.15 mm [3].

The phenomenon of oil absorption is an important issue in the field of deep frying, studied by many researchers. Some of the main factors influencing oil absorption are process conditions such as temperature and time, pretreatments applied, the physicochemical properties of the food, the origin of the oil, its chemical composition, and the initial water content of the food components [55, 56]. Traditional starch‐based coatings gelatinize with heat, forming a more compact structure that prevents the coating from absorbing oil. However, since coatings made with fibrous vegetables do not achieve this gelatinization, the coating may become less cohesive and more porous during frying, which, in some cases, increases oil absorption. Considering that frying temperature and duration are the main process parameters affecting oil uptake [57], the 14.73% oil absorption rate obtained in this study is higher than the literature values. The use of vegetable mixtures rich in dietary fiber, which provide plasticity with heat but do not contain gluten and starch, is crucial for achieving less oil absorption and higher moisture content in the production of crispy chicken. In this context, the process should be adjusted using different vegetable mixtures with investigated dietary fiber content and varying frying temperatures.

### 3.4. Color Properties

Since the liquid coating used in crispy chicken production is the most important parameter affecting the adhesion performance of the dry coating, a nine‐trial model was developed using the Minitab statistical package and RSM to determine the proportions of corn starch, water, and salt to be used. The color measurement values for these nine trial models are presented in Table 5.

**Table 5:** Color measurement values related to liquid coating models used in crispy chicken production^a^.

**Samples**	**L** ^∗^	**a** ^∗^	**b** ^∗^	**C** ^∗^	**h** ^0^
1	80.10 ± 0.141^bcd^	0.10 ± 0.141^ab^	5.70 ± 0.141^c^	5.70 ± 0.141^c^	88.85 ± 1.344^a^
2	81.30 ± 0.424^abc^	0.30 ± 0.213^a^	7.90 ± 0.141^a^	7.90 ± 0.141^a^	88.00 ± 0.283^a^
3	79.25 ± 1.06^cd^	0.15 ± 0.071^ab^	5.40 ± 0.424^c^	5.40 ± 0.424^c^	88.45 ± 0.354^a^
4	82.05 ± 0.91^ab^	0.25 ± 0.071^a^	8.10 ± 0.424^a^	8.10 ± 0.42^a^	88.2 ± 0.283^a^
5	78.60 ± 0.283^d^	0.25 ± 0.071^a^	5.50 ± 0.283^c^	5.50 ± 0.283^c^	87.85 ± 0.636^a^
6	82.30 ± 1.980^ab^	0.30 ± 0.141^a^	7.95 ± 0.212^a^	7.95 ± 0.21^a^	88.00 ± 1.27^a^
7	83.10 ± 0.424^a^	0.00 ± 0.213^ab^	6.80 ± 0.283^b^	6.80 ± 0.283^b^	90.25 ± 2.333^a^
8	80.25 ± 1.202^bcd^	0.10 ± 0.213^ab^	6.65 ± 0.071^b^	6.65 ± 0.071^b^	89.05 ± 2.899^a^
9	82.25 ± 1.344^ab^	−0.10 ± 0.071^b^	7.00 ± 4.950^b^	7.00 ± 4.950^b^	90.70 ± 0.283^a^
Statistical significance	*p* = 0.001^∗∗^	*p* = 0.003^∗∗^	*p* = 0.003^∗∗^	*p* = 0.001^∗∗^	*p* = 0.020^∗^

^a^Means in columns followed by different letter(s) (^abcd^) are sign significantly different.

^∗^Significant differences at *p* < 0.05,  ^∗∗^significant differences at *p* < 0.01, respectively.

In the ANOVA applied to the color measurement values of liquid coating solutions consisting of corn starch, water, and salt, significant differences were detected among the groups for *L*∗, *a*∗, *b*∗, and *C*∗, while no significant difference was observed for *h* ^0^ measurement values between the groups. The detailed content of the selected model, based on the nine samples and the measured viscosity values obtained from the mathematical modeling, is presented in Figure 3. The color measurement values of the raw and cooked chicken breast samples coated with the coating material described in detail in Figure 1, as well as the uncoated chicken breast samples, are presented in Table 6.

**Table 6:** Color properties of stored chicken meat samples.

**Days**	**Samples**	**L** ^∗^	**a** ^∗^	**b** ^∗^	**Δ** **E**
0	BM	57.91 ± 2.90^a^	7.48 ± 1.42^a^	19.96 ± 3.72^a^	0.00
	UCBM	59.07 ± 0.53^b^	5.25 ± 0.57^d^	27.34 ± 0.49^b^	0.00
	CCBM	19.76 ± 10.15^c^	18.24 ± 8.96^e^	18.72 ± 8.79^e^	0.00

3	BM	56.40 ± 1.74^a^	4.42 ± 0.32^bc^	16.28 ± 0.40^a^	5.02^b^
	UCBM	55.09 ± 3.03^b^	3.98 ± 0.83^d^	23.78 ± 0.89^cd^	5.49^b^
	CCBM	35.15 ± 0.98^c^	8.69 ± 1.40^e^	19.70 ± 1.78^e^	18.14^a^

5	BM	55.99 ± 1.01^a^	4.59 ± 0.66^bc^	16.23 ± 1.18^a^	5.09^b^
	UCBM	59.17 ± 2.05^b^	4.18 ± 0.96^d^	23.61 ± 1.38^d^	3.88^b^
	CCBM	34.67 ± 1.65^c^	14.36 ± 2.28^e^	23.21 ± 1.89^e^	16.05^a^

7	BM	54.89 ± 0.91^a^	6.76 ± 1.59^ab^	17.71 ± 0.60^a^	3.83^b^
	UCBM	56.43 ± 0.42^b^	4.40 ± 0.29^d^	24.99 ± 1.24^bcd^	3.64^b^
	CCBM	29.67 ± 3.21^c^	15.35 ± 2.16^e^	20.35 ± 3.64^e^	10.45^a^

9	BM	56.53 ± 1.96^a^	3.46 ± 0.90^c^	17.55 ± 1.62^a^	4.89^b^
	UCBM	57.39 ± 2.90^b^	4.78 ± 0.60^d^	25.30 ± 0.99^bcd^	2.68^b^
	CCBM	33.10 ± 4.92^c^	12.33 ± 1.38^e^	20.66 ± 5.24^e^	14.72^a^

11	BM	55.64 ± 0.60^a^	5.42 ± 0.10^abc^	17.43 ± 0.69^a^	3.97 ^b^
	UCBM	54.44 ± 2.24^b^	5.05 ± 0.32^d^	26.28 ± 0.19^bc^	4.75^b^
	CCBM	28.04 ± 0.39^c^	12.67 ± 1.74^e^	19.66 ± 7.60^e^	10.02^a^

Statistical significance	BM	ns (*p* = 0.432)	*p* = 0.003^∗∗^	ns (*p* = 0.199)	*p* = 0.001
	UCBM	ns (*p* = 0.083)	ns (*p* = 0.184)	*p* = 0.003^∗∗^	*p* = 0.826
	CCBM	*p* = 0.019^∗^	ns (*p* = 0.165)	ns (*p* = 0.941)	*p* = 0.001

*Note:* Means in columns followed by different letter(s) (^abcd^) are sign significantly different.

Abbreviations: BM, chicken breast meat; CCBM, cooked vegetable coated breast meat; UCBM, uncooked vegetable coated breast meat.

^∗^Significant differences at *p* < 0.05,  ^∗∗^significant differences at *p* < 0.01, respectively.

The *L*∗ and *a*∗ values of the chicken breast samples were similar to those of the coated products; however, a decrease in the *L*∗ values was observed during the cooking process due to the burning of vegetables in the coating. Throughout the shelf life, no statistically significant difference was found in the *L*∗ values of the chicken breast, coated raw, and cooked samples between the days, with *p* values of 0.432, 0.083, and 0.019, respectively. Regarding the *a*∗ values, positive values were observed in all samples, indicating that the redness was more prominent than the greenness. A significant difference in the *a*∗ values was observed in the chicken breast during the storage period (*p* = 0.003), whereas no statistically significant difference was found in the coated raw (*p* = 0.184) and cooked (*p* = 0.165) samples. It is likely that the higher *a*∗ value observed in the chicken breast, compared to the coated products, is due to the pink color of the surface of the breast meat. The substantial increase in the *a*∗ values observed in the cooked coated products is interpreted to result from the 11% carrot powder used in the dry coating and the darkening of the carrot powders during cooking. The highest *b*∗ value was naturally found in the coated raw product samples due to the use of 32% yellow‐colored corn semolina in the dry coating. No statistically significant difference was observed in the *b*∗ values of the chicken breast (*p* = 0.199) and the cooked coated product samples (*p* = 0.941) during the shelf life, while a significant difference was detected in the raw coated product samples (*p* = 0.003). As a result of *Δ* *E* calculations, when compared to the reference group BM, a moderate color change was observed in the UCBM group, while a very high color difference emerged in the CCBM group. This value shows that the product has significantly changed visually in the CCBM group. These findings reveal that the color parameters have changed significantly, especially in the CCBM group, and that this change is at a level that can affect consumer perception. When the color measurement values of chicken nuggets coated with coating materials prepared using amaranth, buckwheat, emmer wheat, and mixtures of these flours were examined, a similar trend was observed in our study, where a decrease in *L*∗ values and an increase in *a*∗ and *b*∗ values were recorded after the cooking process [22].

### 3.5. HMF Analysis

In the analysis conducted to determine the HMF levels in raw chicken breast samples coated with the mixture detailed in Figure 2 and in cooked chicken breast samples coated with the same mixture, no HMF was detected in any of the samples. HMF is a furanic compound that forms as an intermediate product during Maillard reactions and caramelization processes. Cooking methods play a significant role in preventing browning, which is strongly related to Maillard reactions and HMF formation [58]. In addition to the cooking method, water activity (aw) values of 0.4 are critical for HMF formation, as several studies in the literature indicate that HMF formation significantly increases beyond this point [59, 60]. In a study on chicken nuggets made with gluten‐free flours such as amaranth, chia, quinoa, and teff, HMF levels were also found to be related to water activity [6]. Furthermore, the literature suggests that adding antioxidant‐rich compounds to meat and its products can reduce the formation of harmful components, while also improving the quality of the meat during frying [61]. The absence of HMF in the vegetable‐coated crispy chicken produced in this study is thought to be due to the high fiber content of the vegetable coating, which reduces the starch content, and the presence of antioxidant compounds in the coating material.

### 3.6. Thermal Properties

Table 7 presents the thermal properties characterized by glass state (Tg) and denaturation temperatures. The denaturation and glass transition temperatures decreased due to the frying process. In terms of storage stability, it was found that the batter material had a slightly negative effect on the thermal properties of the products (*p* > 0.05). It is believed that the free moisture in the coating material composition contributes to this effect. However, the Tp value was found to be quite high compared to battered nuggets obtained with powder mixtures containing different hydrocolloids. The interaction between the moisture in the batter material and the unfrozen moisture in the chicken meat affects ice crystallization depending on the type and concentration of the gelling agent used in the batter material.

**Table 7:** Thermal properties of unfried and fried nuggets.

**Codes**	**Tp (°C)**	**Tg (°C)**
BM	110.0 ± 0.1	−21.1 ± 0.02
UCBM	123.2 ± 0.02	−20.85 ± 0.01
CCBM	118.7 ± 0.01	−27.8 ± 0.01

Abbreviations: BM, chicken breast meat; CCBM, cooked vegetable‐coated breast meat; UCBM, uncooked vegetable‐coated breast meat.

The endothermic peaks in DSC thermograms indicated Tg. The Tg values of nuggets varied from −20.85°C to −27.8°C (Table 7). Tg values of chicken nuggets battered with different biopolymers have been reported as −17.08 ± 0.04 [62], −16.83°C [63] and −16.63°C [64]. The data for fried and nonfried chicken meat, both coated and uncoated with coating material prepared with different amounts of starch and salt, were determined to be lower than previous study findings. Additionally, the batter material increased the glass transition temperature compared to the control sample, but the frying process decreased it (*p* > 0.05). Xue and Ngadi [65] reported that this occurs due to the concentration of the gelling agents in the batter material and the inter‐ and intramolecular interactions of the functional groups they contain. It has also been reported that dramatic changes may occur due to the effect of different molecular structures added to the batter material on the molecular mobility of moisture, depending on the concentration [66]. In addition to directly affecting storage stability, it also indicates that using coating materials with optimum concentration content, which provides a positive effect on flavor and texture, can directly influence storage stability.

As shown in Figure 5, the thermal diagrams indicate distinct transitions for (a) denaturation and (b) the glassy state, supporting the observations presented in Table 7.

Figure 5Thermal diagrams of the chicken nuggets as (a) denaturation and (b) glassy state.

**Figure:**
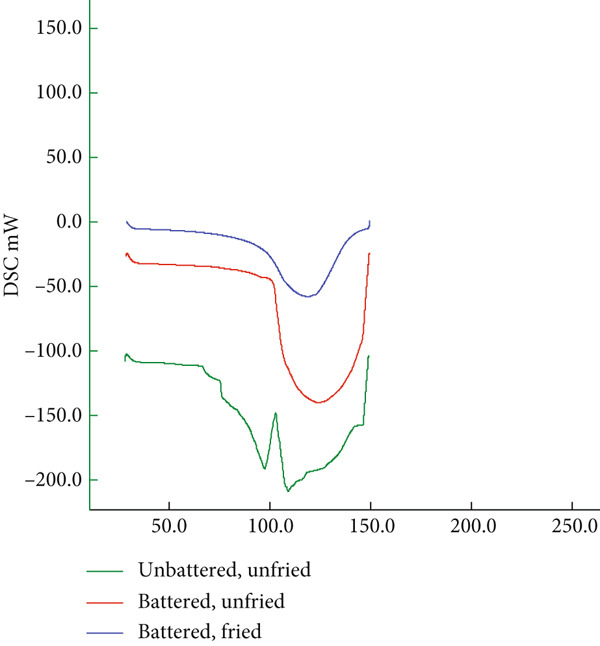
(a)

**Figure:**
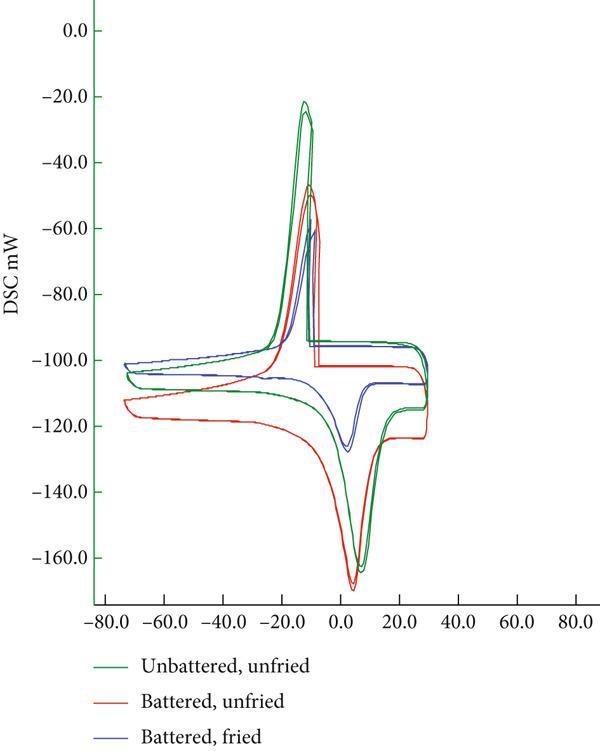
(b)

### 3.7. Texture Profile

The TPA results of vegetable‐coated chicken breasts are presented in Table 8 as cooked (CCBM) and uncooked (UCBM).

**Table 8:** Texture profile analysis (TPA) results of coated uncooked and cooked chicken samples.

**Parameter**	**UCBM**	**CCBM**
Hardness (N)	29.40 ± 14.104	182.524 ± 58.64
Springiness	0.5387 ± 0.244	0.8849 ± 0.056
Cohesiveness	0.4245 ± 0.042	0.7141 ± 0.061
Gumminess	1270.66 ± 580.292	13,032.34 ± 3178.764
Chewiness	735.54 ± 557.379	11,419.54 ± 2289.912
Resilience	0.1904 ± 0.029	0.3987 ± 0.048

Abbreviations: CCBM, cooked vegetable‐coated breast meat; UCBM, uncooked vegetable‐coated breast meat.

Textural properties, which are a key parameter for consumer acceptance of a food product, play a significant role in determining production quality. The texture measurement of meat and meat products relies on the instrumental assessment of sensory attributes such as hardness, elasticity, chewiness, cohesiveness, and juiciness. In this context, the hardness, elasticity, cohesiveness, gumminess, chewiness, and resilience of raw and cooked crispy chicken samples coated with the vegetable powder composition described in detail in Figure 1 were evaluated. These parameters were determined based on the definitions formulated by [67]. In this regard, hardness is defined as the force required to cause a certain deformation in the structure of the food, corresponding to the point where the first compression ends and the release begins. Cohesiveness represents the strength of the bonds forming the structure of the food and is the ratio of the positive force observed during the second compression to the positive force observed during the first compression. Elasticity is defined as the rate at which the food returns to its original state after the deforming force is removed, corresponding to the time interval between the end of the first compression and the beginning of the second compression. Gumminess is the energy required to disintegrate a semisolid food until it is ready to be swallowed, calculated by multiplying the values of hardness and cohesiveness. Chewiness is defined as the energy required to disintegrate solid foods until they are ready to be swallowed and is calculated by multiplying hardness, cohesiveness, and elasticity.

Upon reviewing the TPA results of the raw and cooked crispy chicken samples coated with vegetable powder, it was observed that the hardness values were 2998.72 and 18612.71 g, the cohesiveness values were 0.4245 and 0.7141, the gumminess values were 1270.66 and 13,032.34, the chewiness values were 735.54 and 11,419.54, and the resilience values were 0.1904 and 0.3987, respectively.

The desired texture of the product is highly dependent on the characteristics of the coating materials. It has been noted that the different properties of flours, gums, and starches used for coating can significantly affect the final product’s texture [33]. The variations in texture profile values can be attributed to factors such as product formulation (the amount of water in the formulation increases hardness), cooking methods, process steps, and different meat types [68]. Frying time and temperature also cause changes in the textural properties of the samples. These changes may include a crispy or crunchy outer layer due to reduced moisture, a softer texture due to protein denaturation, and a thicker, more adhesive coating due to starch gelatinization [69].

### 3.8. Microbiological Analysis

**Figure 6:**
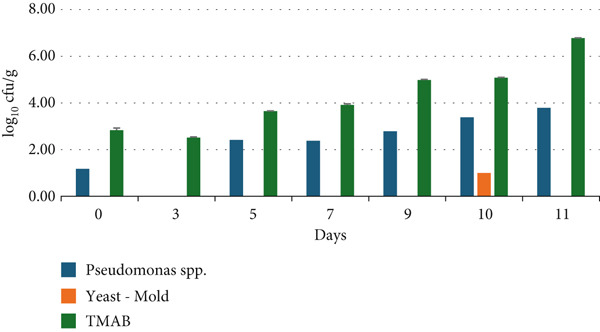
Changes in the microbiological viability (log_10_cfu/g) of chicken breast meat during the storage period.

**Figure 7:**
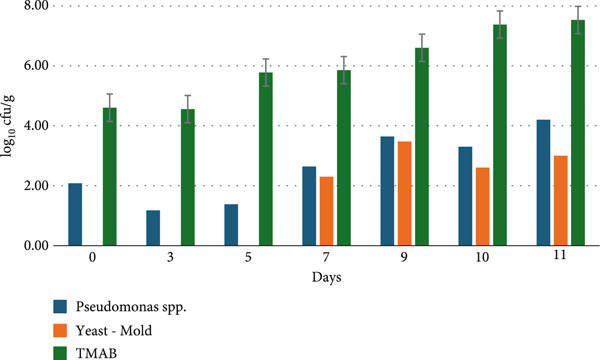
Changes in the microbiological viability (log_10_cfu/g) of chicken breast meat coated with a vegetable mixture during the storage period.

The results of the shelf‐life analysis of raw and cooked chicken breast samples coated with the coating material detailed in Figure 1 are presented in Figures 6 and 7.

Upon examining the results of the 11‐day shelf‐life analysis of chicken breast samples without vegetable powder coating, it was observed that the count of *Pseudomonas* spp. counts ranged from 0.00 to 3.79 log_10_cfu/g, yeast and mold counts ranged from 0.00 to 1.00 log_10_cfu/g, and total aerobic mesophilic bacteria (TAMB) counts ranged from 2.52 to 6.78 log_10_cfu/g, while *Salmonella* spp. and coliform bacteria were not detected. In contrast, for chicken breast samples with vegetable powder coating, the 11‐day shelf‐life analysis results showed that *Pseudomonas* spp. counts ranged from 1.18 to 4.20 log_10_cfu/g, yeast and mold counts ranged from 0.00 to 3.48 log_10_cfu/g, and TAMB counts ranged from 4.56 to 7.53 log_10_cfu/g, while *Salmonella* spp. and coliform bacteria were also not detected. In general, it can be noted that the microbial load increased over time as the storage period progressed.

According to international food safety standards, the presence of *Salmonella* spp. in chicken meat is strictly prohibited, with a zero‐tolerance policy applied to 25 g of sample. For coliforms, the acceptable limit is generally set at 50 cfu/g for cooked chicken products and up to 5000 cfu/g for raw poultry. While specific regulatory limits for *Pseudomonas* spp. are not clearly defined, their presence in ready‐to‐eat or cooked meat products is considered undesirable due to their spoilage potential. Acceptable levels of *yeasts and molds* are typically ≤ 10 cfu/g in cooked meat products, while for raw products, some regulations allow up to 10^3^ cfu/g. Regarding *TMAB*, limits vary based on the processing status of the product: ≤ 1000 cfu/g is generally accepted for cooked poultry, whereas for raw meat, values up to 10^5^–10^7^ cfu/g are considered acceptable, depending on the specific standard applied. These thresholds serve as critical reference points for evaluating the microbial safety and shelf‐life performance of vegetable powder–coated chicken meat products [70].

Coliform bacteria, which belong to the *Enterobacteriaceae* family, are facultative anaerobic, gram‐negative, nonspore‐forming, rod‐shaped bacteria that produce gas and acid from lactose within 48 h at 35°C–37°C. Due to their widespread presence in the intestines and the environment (e.g., soil, plants), they are commonly used as sanitation indicators in the food industry. Therefore, the presence of a high level of coliform microorganisms in meat and meat products suggests inadequate hygienic practices during slaughter, processing, storage, or sale [71]. In our study, the fact that no coliform bacteria or *Salmonella* spp. were detected in the samples analyzed, even on the last day of storage, indicates that the production and storage conditions adhered to hygienic precautions [72]. Yeasts and molds are another group of microorganisms that contribute to the spoilage of chicken meat. These organisms can grow over a wide pH range (pH 2–9), at storage temperatures (10°C–35°C), and water activity (0.85 or higher). They can also utilize proteins, lipids, organic acids, and other complex carbohydrates. In our study, yeast and mold growth was observed after the seventh day, particularly in the coated chicken breast samples, indicating that further storage would likely lead to continued microbial growth and a decline in nutritional quality. Although previous studies have reported higher yeast and mold counts compared to those observed on the first day in our samples [35, 73], the initial microbial load of the vegetables used in the coating material should be carefully considered, and appropriate preventive measures must be taken.

*Pseudomonas* is an aerobic, gram‐negative bacterium commonly found in soil. It can grow well across a temperature range from 2°C to 35°C and is often found in chilled food products as well as foods prepared at room temperature [73]. In a microbiological analysis aimed at detecting *Pseudomonas* spp. in samples collected from four different slaughterhouses, the results indicated that *Pseudomonas* spp. counts varied between 4.62 and 5.06 log_10_cfu/g [74].

The TMAB counts are an important criterion in providing information about the processing and storage conditions, product safety, and quality of meat and meat products. The values found in similar studies show consistency with the current research. TMAB are widely recognized not only as indicators of the current microbial load in chicken meat but also as reliable markers of processing hygiene and storage quality. For instance, Abdullah et al. [75] reported that TMAB counts increase during cold storage, reflecting the microbial spoilage of meat. Elevated TMAB levels have been directly associated with a decline in sensory quality, including off‐odors, discoloration, and textural degradation [76]. Moreover, the control of TMAB is considered a fundamental criterion for evaluating the hygienic effectiveness of processing and storage practices. High TMAB counts may indicate deficiencies in these critical control points. Therefore, TMAB serves as an indispensable parameter in assessing the microbial safety and overall quality of chicken meat [76–78]. In the present study, higher bacterial counts were also detected in vegetable‐coated samples. It is believed that the initial microbial load of the vegetables used as coating material contributed to this situation, and improvements regarding the initial microbial loads of the vegetables should be made to prevent adverse long‐term effects on the nutritional composition of the chicken breasts.

The microbial contamination levels on food contact surfaces of the equipment before operation were not significantly lower than those after processing [79]. Most bacteria require a water activity (aw) value above 0.85 in order to multiply. In particular, TMAB tend to proliferate rapidly when the water activity (a_a_) exceeds approximately 0.7. The fibrous structure of vegetable powders contributes to moisture retention on the surface, thereby increasing water activity and creating a favorable environment for microbial growth [80]. The combination of these factors may lead to higher TMAB counts in vegetable powder–coated chicken meat compared to the control group. During the development phase, parameters such as the source of the vegetable powder, pretreatment processes, coating formulation, and production hygiene should be thoroughly examined and optimized.

### 3.9. Sensorial Properties

Panelists consisting of female (number of people) and male (number of people) employees of Hastavuk Karapürçek, Turkey facilities, aged between 28 and 35 and 33 were selected through a pretest on taste sensitivity. Panelists were semieducated panelists who received training on eating awareness and taste and TPA. Panelists participating in the study had the necessary ethics committee permission documents. The sensory evaluation conducted by a group of volunteer panelists on a 10‐point scale is presented in Table 9. Upon reviewing the sensory evaluation results, the criteria for appearance, odor, general taste, coating quality, and greasiness scored between very good (8) and excellent (9), while crunchiness was rated between good (7) and very good (8). The overall liking score in the sensory evaluation was above 8, with the lowest score observed for crunchiness at 7.84. It was interpreted that the relatively lower score for the crunchiness criterion may be due to the fact that panelists were accustomed to the crunchiness imparted by ingredients such as wheat and breading used in commercially available yellow‐coated products, whereas the vegetable powder formulation used in our study did not achieve the same effect.

**Table 9:** The evaluation form presented to the sensory panelists.

**Sensory evaluation form** **Panelist name:** **Date:** **Sample code:** **Instructions:** Please evaluate each attribute of sample based on your perception, using a scale from 1: Extremely poor to 10: Excellent. Write the score that best reflects your opinion.			
**Attribute**	**Definition**		**Score (1–10)**
Appearance:	Overall visual quality, shape, and surface uniformity		
Odor:	Pleasantness, intensity, and freshness of smell		
General taste:	Balance, intensity, and overall palatability of flavor		
Coating quality:	Uniformity, adhesion, and visual appeal of the coating layer		
Greasiness:	Perceived oiliness or fat residue on taste and mouthfeel		
Crunchiness:	Perceived crispiness and firmness during bite		
Comments: Observations:			
Scale guide:			
1: Extremely poor	5: Moderate	8: Very good	9–10: Excellent

**Figure 8:**
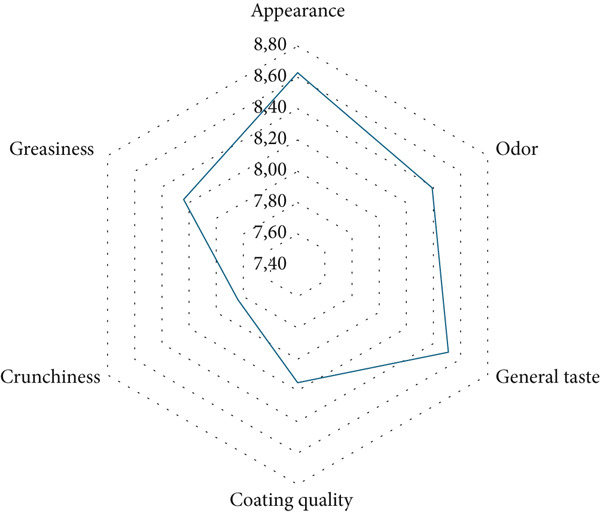
Spider web diagram of the sensory evaluation results for vegetable powder–coated crispy chicken sample.

The spider web graph depicting the sensory evaluation results of the vegetable‐coated crispy chicken samples is presented in Figure 8. It is evident that all vegetable‐coated crispy chicken samples received acceptable scores, which can be interpreted as a promising indication of the potential for the recipe applied in this study.

Pursell et al. [81] reported that the oxidation level used in the production of chicken nuggets using oxidized starch did not change the gelatinization temperature of most starches, and there was no difference in all sensory qualities between treatments and between wheat flour control and treatments partially modified with oxidized starches [82]. De Carvalho et al. [82] reported that in the sensory evaluation of nugget samples formulated with gluten‐free coating materials, the sample with modified tapioca starch added received the highest overall acceptability score (7.3) and the sample with wheat flour added received the lowest score by untrained panelists [82]. Another study reported no difference in acceptability between gluten‐free and gluten‐containing nuggets (*p* = 0.14). The researchers reported that rice and chickpea flour were viable substitutes for wheat flour in chicken nugget batter [83]. The overall acceptability especially crunchiness of the gluten‐free nuggets produced in our study was scored higher than the results obtained with chickpea flour, oxidized starch derivatives, and rice flour used in studies by other researchers.

## 4. Conclusion

The preparation of gluten‐free meat products using cereal sources and dried vegetables is an emerging area for researchers and the industry. The inclusion of vegetables not only enhances the nutritional value of meat products, which are typically low in dietary fiber, but also allows the development of functional foods. Among cereal sources, wheat is commonly used as a coating material; however, it does not cater to consumers with gluten sensitivities due to its gluten protein content. In this study, corn fiber–containing precoating formulations were developed as an alternative to wheat protein, and gluten‐free dry coating formulations enriched with high protein content and fiber were produced using dried vegetable mixtures (oyster mushrooms, spinach, carrots, onions, and corn semolina) and buckwheat flour. Additionally, the liquid coating was optimized with a gluten‐free corn flour mixture to produce crispy chicken. The aim was to develop a healthy product with high coating efficiency, desirable taste, a good texture profile, minimal deformation after cooking, and high fiber content. The findings related to the product’s physical, sensory, and textural properties suggest that it may meet consumer demand. This study has also some limitations, such as the following:

− Accessing dried vegetable powders is challenging because some powders are only available during the seasons when the vegetable is produced. Therefore, planned and sustainable supply in industrial production can be challenging.
− Vegetable powders, despite being processed through the same grinder, cannot be ground to a uniform size due to their inherent structure and therefore may not be distributed homogeneously when coating the chicken.
− Wheat starch–based industrial liquid coatings adhere to both the meat and the dry coating, and separation of the coating does not occur during cooking. However, separation can be observed after cooking with coatings such as gluten‐free corn starch because the amylose and amylopectin ratios, which facilitate gelation, are different compared to wheat starch. Ensuring better adhesion of the coating could be a separate topic for future research.

Despite some limitations mentioned above, this study has significant potential for application in the food industry, not only providing an alternative nugget product that can meet consumer demand but also serving as a reference for improving the quality characteristics of gluten‐free functional food processing with dietary fiber. It may also provide guidance for future studies into developing egg‐coated products with the aim of reducing or eliminating eggs as an allergen source.

## Conflicts of Interest

The authors declare no conflicts of interest.

## Funding

The study is supported by the HasTavuk Co. R&D Department.

## Data Availability

The data that support the findings of this study are available from the corresponding author upon reasonable request.
